# Impact of the 99DOTS digital adherence technology on tuberculosis treatment outcomes in North India: a pre-post study

**DOI:** 10.1186/s12879-023-08418-2

**Published:** 2023-07-31

**Authors:** Amy Z. Chen, Ravinder Kumar, R. K. Baria, Pramod Kumar Shridhar, Ramnath Subbaraman, William Thies

**Affiliations:** 1 Everwell Health Solutions, Bangalore, Karnataka India; 2 World Health Organization, Himachal Pradesh, Shimla, India; 3 Directorate of Health Services, Himachal Pradesh, Shimla, India; 4 Maharishi Markandeshwar Medical College & Hospital, Kumarhatti-Solan, Himachal Pradesh India; 5 Department of Public Health and Community Medicine and Center for Global Public Health, Tufts University School of Medicine, Boston, MA US

**Keywords:** Tuberculosis, Medication adherence, Digital adherence technologies, mHealth

## Abstract

**Background:**

99DOTS is a cellphone-based digital adherence technology. The state of Himachal Pradesh, India, made 99DOTS available to all adults being treated for drug-sensitive tuberculosis (TB) in the public sector in May 2018. While 99DOTS has engaged over 500,000 people across India, few studies have evaluated its effectiveness in improving TB treatment outcomes.

**Methods:**

We compared treatment outcomes of adults with drug-sensitive TB before and after Himachal Pradesh’s 99DOTS launch using data from India’s national TB database. The pre-intervention group initiated treatment between February and October 2017 (*N* = 7722), and the post-intervention group between July 2018 and March 2019 (*N* = 8322). We analyzed engagement with 99DOTS and used multivariable logistic regression to estimate impact on favorable treatment outcomes (those marked as cured or treatment complete).

**Results:**

In the post-intervention group, 2746 (33.0%) people called 99DOTS at least once. Those who called did so with a wide variation in frequency (< 25% of treatment days: 24.6% of callers; 25–50% of days: 15.1% of callers, 50–75% of days: 15.7% of callers; 75–100% of days: 44.6% of callers). In the pre-intervention group, 7186 (93.1%) had favorable treatment outcomes, compared to 7734 (92.9%) in the post-intervention group. This difference was not statistically significant (OR = 0.981, 95% CI [0.869, 1.108], *p* = 0.758), including after controlling for individual characteristics (adjusted OR = 0.970, 95% CI [0.854, 1.102]).

**Conclusions:**

We found no statistically significant difference in treatment outcomes before and after a large-scale implementation of 99DOTS. Additional work could help to elucidate factors mediating site-wise variations in uptake of the intervention.

## Background

Tuberculosis (TB) is one of the leading infectious causes of death globally, with risks of death, relapse, and acquired drug resistance increased by poor adherence to medications [1, 2]. India’s National Tuberculosis Elimination Program (NTEP), alongside other countries’ programs, has relied on facility-based and community-based directly observed therapy (DOT) to support treatment adherence. However, while DOT has been successful in increasing engagement with health workers, there are concerns about its effectiveness [3] as well as the burdens it places on people being treated for TB [4–6] as well as on health systems [7].

Digital adherence technologies (DATs) have recently emerged as a tool to enable alternative approaches to support adherence [8, 9]. 99DOTS is one such low-cost, cellphone-based technology [10]. For each dose, people on treatment call an unpredictable toll-free number revealed when they dispense drugs from an innovative medication packaging. This call data is used to construct a dosing record that health workers can access on a website or mobile application. People who do not call are highlighted for follow-up; optionally, 99DOTS can be configured to send electronic reminders to people on treatment and alerts to care providers. To date, 99DOTS has engaged over 500,000 people in India [11] and has been deployed in 7 additional countries in Asia and Africa.

Studies on the usability [10, 12, 13] and accuracy [14–16] of 99DOTS have informed its adoption, but relatively few studies have evaluated the technology’s effectiveness in improving TB treatment outcomes [17–19]. One high-quality stepped-wedge cluster randomized trial conducted in Uganda found that 99DOTS did not improve treatment outcomes in the overall population of people being treated for TB [19]. Although the per protocol analysis of the trial results showed a statistically significant improvement in treatment outcomes among people in the intervention period who engaged with 99DOTS, this finding may have been influenced by selection bias. Only about half of people in the intervention period engaged with the 99DOTS, due to barriers in reporting (e.g., lack of cellphone access) and discretion exercised by healthcare providers in provision of the technology.

The impact of 99DOTS on TB treatment outcomes may be context specific. Its effectiveness may vary based on health system factors, such as capacity for new interventions and disposition of healthcare providers towards the technology, or end user factors, such as cellphone literacy or TB-related stigma. As such, further research is needed to understand the impact of 99DOTS on TB outcomes in different settings.

In India, the state of Himachal Pradesh made 99DOTS available to all adults with drug-sensitive TB in May 2018. In this study, we aimed to assess the effect of the state’s adoption of 99DOTS on treatment outcomes for people with drug-sensitive TB treated in the public sector by comparing the favorable treatment outcome rate before and after the 99DOTS launch.

## Methods

### Study setting

Himachal Pradesh is a state located in northern India. It is a mountainous region, and mostly rural with several urban centers. It has 12 districts, which are further divided by NTEP into 74 TB Units (TUs), each of which is assigned a Senior Treatment Supervisor responsible for overseeing the TB program at the health facilities in the TU.

Himachal Pradesh first used 99DOTS in November 2016 as part of NTEP’s initial nationwide pilot of the technology for all adults with HIV being treated for drug-sensitive TB. In February 2017, five states including Himachal Pradesh adopted daily fixed dose combination (FDC) drugs to replace a thrice-weekly intermittent regimen, after which they each launched 99DOTS for all adults with drug-sensitive TB. Himachal Pradesh’s launch was in May 2018.

### Adherence support during the pre-intervention period

Prior to May 2018, nearly all people treated for TB in the public sector in Himachal Pradesh were to receive medications via DOT, either at a nearby health facility or from community-based treatment supporters (including but not limited to Accredited Social Health Activists, or ASHAs). The details of how DOT was implemented for daily-dose medications may have relied on state and local discretion during the study period. When formal NTEP guidelines were first issued (in 2020), they prescribed daily observation of doses in the intensive phase and at least twice-weekly observation in the continuation phase [20]. Some doses may have been given to take unobserved under special circumstances, including weekends and holidays.

Himachal Pradesh first used 99DOTS in November 2016 as part of NTEP’s initial nationwide pilot of the technology for all adults with HIV being treated for drug-sensitive TB. However, there are very few people with HIV being treated for TB in Himachal Pradesh: in 2017, 74 of 13,889 people notified with TB were recorded as being HIV positive [21]. Hence, only a very small fraction of the before group would have been exposed to the prior pilot of 99DOTS.

### Adherence support during the post-intervention period with 99DOTS

Himachal Pradesh’s state TB program aimed to enroll in 99DOTS only adults with drug-sensitive TB who had a need for the technology and would be able to use it, as per provider discretion. Specifically, state-level guidelines stated that people eligible for 99DOTS could include those whose professions required frequent travelling, for whom DOT centers were inconvenient, or who had concerns of stigma-related issues. Eligible users also needed to have a mobile connection, not have an alcohol use disorder, and “assure strict compliance to the instructions after detailed counseling about importance of treatment adherence” [22]. With 99DOTS, a person on treatment received refills of their medicines either from a facility or an ASHA worker, with refill frequencies ranging from once a week to once a month, varying by each facility’s or provider’s practices and the person’s treatment stage and convenience. People who did not use 99DOTS continued to receive their medication via DOT as before.

99DOTS was originally managed using a 99DOTS-specific online website or Android app. In late September 2018, NTEP upgraded Ni-kshay, its digital platform for end-to-end management of tuberculosis, which included the integration of 99DOTS [23]. During the study period, in India, providers could view 99DOTS data in Ni-kshay and see an auto-updated list of people with low adherence as reported by 99DOTS; the 99DOTS system did not send reminders or notifications (to either providers or end users) via SMS or other means. While the upgrade of Ni-kshay also brought minor changes in the interfaces used by health workers to monitor and assign treatment outcomes, the programmatic protocols for categorizing and recording such outcomes remained unchanged. Paper-based treatment cards were also used to record outcomes throughout both periods.

An additional programmatic change during the post-intervention period was the introduction of direct benefit transfers. These were introduced in April 2018 under the Ni-kshay Poshan Yojana scheme, where people are to receive ₹500 for each month for nutritional support while on treatment for TB [21].

### Study subjects and data sources

We obtained case records from Ni-kshay. For the pre-intervention group, we included records for people who initiated treatment between 1 February 2017, when the program adopted daily FDC drugs, and 31 October 2017. This group would finish a standard 24-week regimen by the end of March 2018, allowing a one-month buffer before the May 2018 launch of 99DOTS in case of delayed ending of treatment. For the post-intervention group, we included records for those who initiated treatment between 1 July 2018 and 31 March 2019. While 99DOTS was intended to launch statewide in May 2018, we designate the post-intervention period as starting in July to allow for a two-month buffer for any potential variations in the time needed to launch and stabilize 99DOTS across different locations. The records included personal demographics, disease and treatment details, and 99DOTS calling data. Records from people treated for new, treatment-naïve, drug-sensitive TB aged 18 years and older in the public sector in Himachal Pradesh were included in the study. Records identified as likely duplicate or previously treated were excluded,^1^ as well as records missing treatment outcome data. We considered treatment outcomes entered as “not evaluated” as missing. We defined a “favorable outcome” as those recorded as “cured” or “treatment complete,” whereas an “unfavorable outcome” was those recorded as “died,” “lost to follow up,” “treatment regimen changed,”^2^ or “treatment failure”.

### Statistical analysis

Individuals’ characteristics were described using frequencies and percentages. Pre- and post-intervention groups were compared using *t*-tests for continuous variables and χ^2^ tests for categorical variables. In the post-intervention group, 99DOTS calling data was analyzed to identify who called 99DOTS at least once, as this indicated exposure to the intervention and a minimal ability and willingness to engage with it; of people who never called, we do not have data on what fraction were eligible for the intervention. Demographic and clinical characteristics were compared between people who never called and those who did. For people who called, the percentage of days they called 99DOTS while on treatment was summarized with a mean and histogram.

Treatment outcomes were described using frequencies and percentages. Unadjusted and multivariable logistic regressions were performed to estimate the difference in favorable treatment outcome rate between pre- and post-intervention groups. The multivariable logistic regression controlled for age, gender, and site of disease, and included fixed effects for districts and random effects for TUs; records missing any of these variables were excluded. The variables of age, gender, and site of disease were selected based on (i) availability and completeness in the dataset, and (ii) evidence that they are linked with TB treatment outcomes in India [24–27]. HIV status was not used because of the small number of people recorded as HIV positive. Within the post-intervention group, a χ^2^ test was used to compare the favorable treatment outcome rate between people who never called 99DOTS and those who did, and an unadjusted logistic regression was used to compare the favorable treatment outcome rate across different frequencies of calling (among those who called at least once).

All statistical analyses were performed using Stata/MP 13.0 (StataCorp, College Station, TX, USA).

## Results

Sixteen thousand nine hundred sixty treatment records were retrieved from Ni-kshay on 1 August 2020 for people with new, drug-sensitive TB aged 18 and older who initiated treatment within the study pre- or post-intervention group time periods. Of these, 696 were excluded as likely duplicate or previously treated records. 220 records (99 pre-intervention, 121 post-intervention) were further excluded due to missing treatment outcome data; missingness did not differ significantly between pre- and post-intervention groups (*p* = 0.356, χ^2^ test). 16,044 records were included for most analyses: 7722 records for people treated before and 8322 records for people treated after 99DOTS adoption. The multivariable logistic regression further excluded 157 records that were missing any of the variables used: 156 for missing data on site of disease and 1 for missing data on TU.

Demographic and clinical characteristics of people in the pre- and post-intervention groups are listed and compared in Table 1. Gender, HIV status, and site of disease differed significantly at the 0.05 level.

**Table 1:** Characteristics of study groups

Characteristic	Pre-intervention group (*n* = 7722)		Post-intervention group (*n* = 8322)		*p*-value	Post-intervention group				
						Never called (*n* = 5576)		Called 99DOTS (*n* = 2746)		*p-value*
Age* (mean, SD)	41.9	(17.9)	42.4	(18.3)	0.107	42.8	(18.3)	41.5	(18.1)	0.002
Gender*										
Male (n, %)	4946	(64.1%)	5088	(61.1%)		3393	(60.9%)	1695	(61.7%)	
Female (n, %)	2773	(35.9%)	3233	(38.8%)		2183	(39.1%)	1050	(38.2%)	
Transgender (n, %)	3	(0.0%)	1	(0.0%)	< 0.001	0	(0.0%)	1	(0.0%)	0.265
HIV status										
Not recorded (n, %)	7681	(99.5%)	8252	(99.2%)		5549	(99.5%)	2703	(98.4%)	
Positive (n, %)	41	(0.5%)	70	(0.8%)	0.018	27	(0.5%)	43	(1.6%)	< 0.001
Site of disease*										
Pulmonary (n, %)	5181	(67.1%)	5180	(62.2%)		3535	(63.4%)	1645	(59.9%)	
Extrapulmonary (n, %)	2497	(32.3%)	3030	(36.4%)		2039	(36.6%)	991	(36.1%)	
Unknown (n, %)	44	(0.6%)	112	(1.3%)	< 0.001	2	(0.0%)	110	(4.0%)	< 0.001

Demographic and clinical characteristics of people in the study pre- and post-intervention groups, and of those in the post-intervention group who did and did not call 99DOTS

*p*-values are reported for t-tests for age and χ^2^ tests for all other characteristics. Asterisks (*) indicate variables used in the multivariable logistic regression. *HIV* Human immunodeficiency virus

Of the 8322 people in the post-intervention group, 5576 (67.0%) never called 99DOTS and 2746 (33.0%) called 99DOTS at least once. In the pre-intervention group, 10 people out of 7722 called 99DOTS due to prior initiation of 99DOTS in the HIV program (November 2016); however, no people in the pre-intervention group called 99DOTS after the general statewide launch (May 2018), confirming that the one-month buffer period was sufficient to isolate them from the intervention studied.

**Fig. 1:**
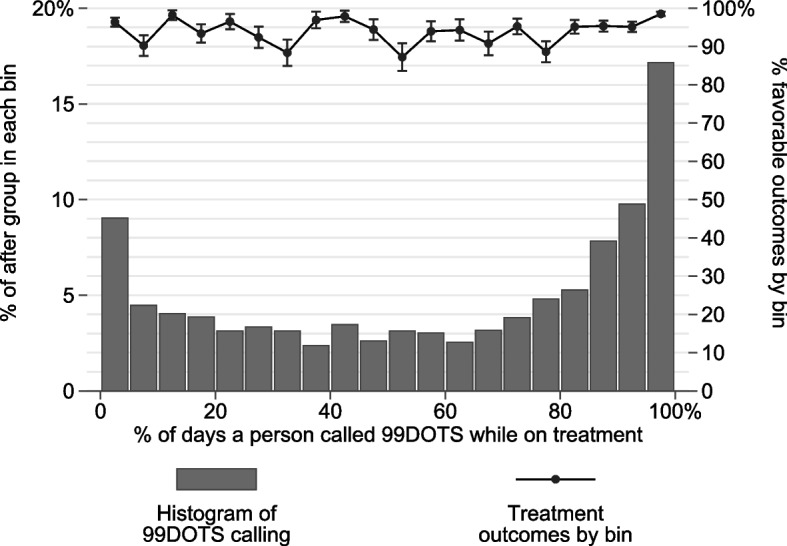
Histogram of 99DOTS calling and treatment outcomes by bin. Histogram of the percentage of days calling 99DOTS while on treatment for people in the post-intervention group who called 99DOTS at least once. Secondary axis illustrates the percentage of people in each bin who had favorable treatment outcomes; bars denote standard errors

Individuals’ characteristics are listed and compared by 99DOTS calling in Table 1. People who called 99DOTS were on average 1.3 years younger, and were more often recorded as HIV positive by 1.1 percentage points, but did not differ significantly by gender. There was also a significant difference in site of disease, owing to differences in the number of unknown (missing) entries; if missing data are excluded, the difference between pulmonary and extrapulmonary is not significant. People who called 99DOTS did so on an average of 58.5% of days during treatment, though the distribution (Fig. 1) shows that many people called either very few times or almost every day (< 25% of days: 24.6% of callers; 25–50% of days: 15.1% of callers; 50–75% of days: 15.7% of callers; 75–100% of days: 44.6% of callers).

**Fig. 2:**
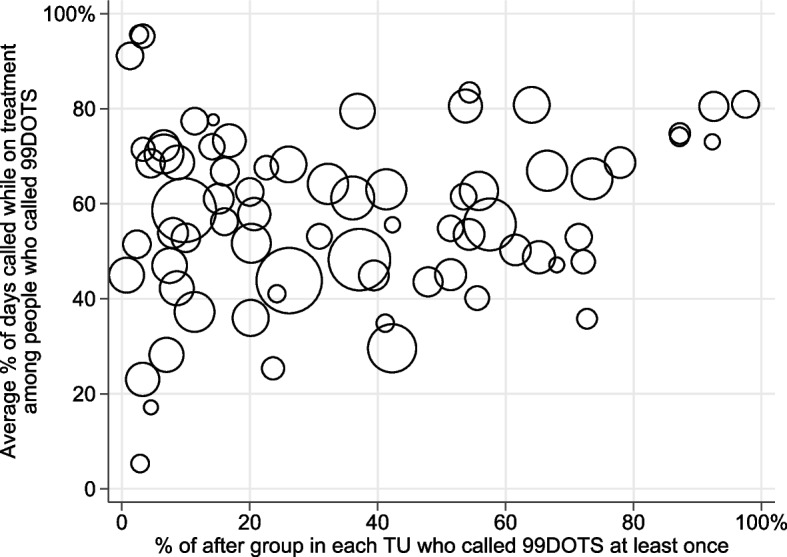
How engagement with 99DOTS varies across sites. Scatter plot of each TU’s percentage of the post-intervention group who called 99DOTS at least once against the average percentage of days calling 99DOTS while on treatment. TU = Tuberculosis Unit, a sub-district administrative unit in India’s National TB Elimination Program. Size of the circle marker corresponds to the size of the post-intervention group in each TU, ranging from 7 to 474 people

There was high variability in 99DOTS uptake by site of treatment (Fig. 2). Across the 74 TUs, there was a wide spread of the proportion of people in the post-intervention group in each TU who had called 99DOTS at least once, ranging from 0.0% to 97.5% of the TU. There was also a wide spread in the average percentage of days called during treatment among people who called 99DOTS, spanning from 5.3% to 95.6%.

**Fig. 3:**
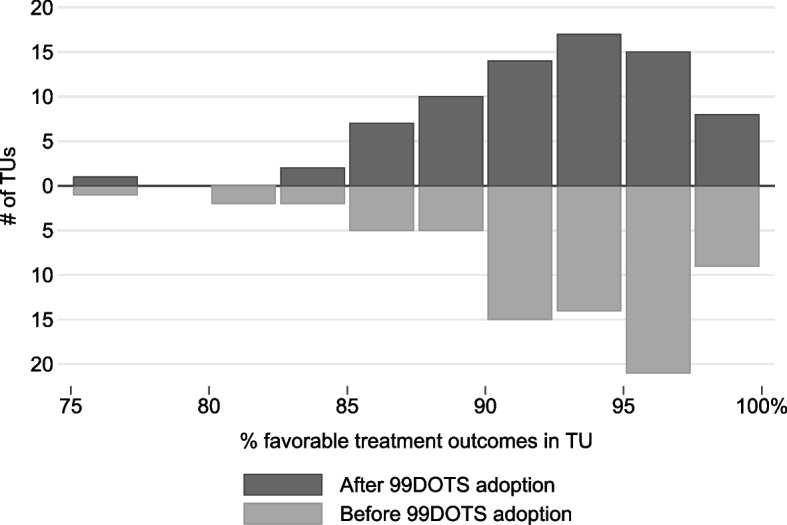
How treatment outcomes vary across sites, before and after the intervention. Histogram of the rates of favorable treatment outcomes by TU, drawn separately for before 99DOTS adoption (below the line) and after 99DOTS adoption (above the line). TU = Tuberculosis Unit

Treatment outcomes for the pre- and post-intervention groups are listed in Table 2. The pre-intervention group had 93.1% (7186/7722) favorable outcomes, and the post-intervention group 92.9% (7734/8322) favorable outcomes. In both groups, a majority of the unfavorable outcomes were death, followed by loss to follow up, treatment regimen change, and treatment failure. The rate of favorable outcomes varied considerably across TUs (Fig. 3).

**Table 2:** Treatment outcomes

	Pre-intervention group (*n* = 7722)		Post-intervention group (*n* = 8322)		Never called (*n* = 5576)		Called 99DOTS (*n* = 2746)	
Treatment outcome	n	(%)	n	(%)	n	(%)	n	(%)
Favorable outcome	7186	(93.1%)	7734	(92.9%)	5131	(92.0%)	2603	(94.8%)
Cured	3398	(44.0%)	3492	(42.0%)	2192	(39.3%)	1300	(47.3%)
Treatment complete	3788	(49.1%)	4242	(51.0%)	2939	(52.7%)	1303	(47.5%)
Unfavorable outcome	536	(6.9%)	588	(7.1%)	445	(8.0%)	143	(5.2%)
Died	302	(3.9%)	393	(4.7%)	296	(5.3%)	97	(3.5%)
Lost to follow up	137	(1.8%)	125	(1.5%)	102	(1.8%)	23	(0.8%)
Treatment regimen changed	57	(0.7%)	41	(0.5%)	26	(0.5%)	15	(0.5%)
Treatment failure	40	(0.5%)	29	(0.3%)	21	(0.4%)	8	(0.3%)

Aggregate treatment outcomes for the study pre- and post-intervention groups, and for those in the post-intervention group who called or did not call 99DOTS

Results from logistic regressions comparing favorable treatment outcomes between the pre- and post-intervention groups are detailed in Tables 3 and 4. Unadjusted logistic regression (Table 3) found no significant difference in favorable treatment outcomes between groups (odds ratio (OR) = 0.981, 95% confidence interval (CI) [0.869, 1.108], *p* = 0.758). The multivariable logistic regression (Table 4) also found no significant difference between groups (adjusted OR = 0.970, 95% CI [0.854, 1.102], *p* = 0.643). Two variables (age and gender) were found to have a significant association with treatment outcomes across pre- and post-intervention groups. Older people were less likely to have a favorable outcome (adjusted OR = 0.961, 95% CI [0.958, 0.965], *p* < 0.001), and men were less likely to have a favorable outcome (adjusted OR = 0.778, 95% CI [0.677, 0.894], *p* < 0.001). Some districts also performed more favorably than others, as detailed in Table 4.

**Table 3:** Results of unadjusted logistic regression

	Unadjusted logistic regression		
Variable	OR	[95% CI]	*p*-value
Pre/post 99DOTS launch			
Pre-intervention	(reference)		
Post-intervention	0.981	[0.869,1.108]	0.758
N	16,044		

Results of unadjusted logistic regression for favorable treatment outcome on whether a person was treated before or after the launch of 99DOTS. *OR* Odds ratio, *CI* Confidence interval

**Table 4:** Results of multivariable logistic regression

	Multivariable logistic regression		
Variable	Adjusted OR	[95% CI]	*p*-value
Pre/post 99DOTS launch			
Pre-intervention	(reference)		
Post-intervention	0.970	[0.854,1.102]	0.643
Age	0.961	[0.958,0.965]	< 0.001
Gender			
Female or transgender	(reference)		
Male	0.778	[0.677,0.894]	< 0.001
Site of disease			
Pulmonary	(reference)		
Extrapulmonary	1.133	[0.984,1.305]	0.083
District			
District 1	(reference)		
District 2	0.623	[0.382,1.016]	0.058
District 3	1.038	[0.320,3.365]	0.951
District 4	1.049	[0.623,1.766]	0.857
District 5	1.205	[0.771,1.883]	0.413
District 6	1.248	[0.566,2.752]	0.583
District 7	1.294	[0.767,2.182]	0.334
District 8	1.312	[0.804,2.142]	0.277
District 9	1.342	[0.785,2.293]	0.282
District 10	1.581	[0.914,2.736]	0.102
District 11	1.634	[0.918,2.908]	0.095
District 12	1.655	[1.038,2.637]	0.034
N	15,887		

Results of multivariable logistic regression for favorable treatment outcome on whether a person was treated before or after the launch of 99DOTS. *OR* Odds ratio, *CI* Confidence interval

Within the post-intervention group, people who never called 99DOTS had 92.0% (5131/5576) favorable treatment outcomes, while people who called at least once had 94.8% (2603/2746) favorable outcomes (Table 2). The difference in proportion is statistically significant (*p* < 0.001, χ^2^ test). Among people who called at least once, there is not a statistically significant association between frequency of calling and favorable treatment outcomes (unadjusted logistic regression, OR = 1.49, 95% CI [0.91, 2.4], *p* = 0.108). Figure 1 illustrates the rate of favorable treatment outcomes across different frequencies of calling.

## Discussion

In this study of a rollout of 99DOTS in a North Indian state, we found no significant difference in favorable treatment outcomes between pre- and post-intervention groups. Our observational study leverages administrative data from an at-scale deployment, using a quasi-experimental pre-post design to provide a rigorous assessment of the potential impact of 99DOTS on TB treatment outcomes. While the proportion of people with favorable treatment outcomes in the post-intervention group was greater for people who called 99DOTS than those who did not, due to the non-random uptake of 99DOTS, we cannot determine whether usage of 99DOTS caused this difference.

The setting of the study is important in contextualizing this finding. Himachal Pradesh’s state TB program has consistently had higher favorable treatment outcome rates compared to India overall [21, 23], and had robust baseline implementation of DOT. A high-performing program may be able to manage a higher-quality implementation of 99DOTS; however, there may have been little room for additional improvement in treatment adherence and subsequent treatment outcomes. Studies in settings with worse baseline treatment outcomes or less rigorous baseline adherence support may observe different impacts.

Our study found partial use of 99DOTS, with 33.0% of the post-intervention group having ever called 99DOTS. This does not necessarily represent the proportion of people who were given their drugs in 99DOTS sleeves and instructed to call 99DOTS, as some such people may have never called: for example, in a large-scale Mumbai deployment, 11% of people enrolled in 99DOTS never called [10]. Among people who did call, there was high variability in how often they called, also reported for Mumbai [10]. While the usage of 99DOTS here may seem low, others have noted that 99DOTS should not be viewed as a blanket solution, but rather one of many adherence support methods that may only work for some people [8, 12].

Important questions remain in understanding personal and implementation factors that could influence whether and how frequently people engage with 99DOTS. Of demographic characteristics we could observe, there are only minor differences between groups that called and never called. People who called were on average 1.3 years younger, perhaps reflecting higher rates of household phone access in middle-aged groups compared to older groups in India [28]. They were also more often recorded as HIV positive by 1.1 percentage points, perhaps related to greater maturity of 99DOTS implementation in the TB-HIV program, which launched about 1.5 years before the launch for all adults with drug-sensitive TB. It bears emphasizing that our data only speak to engagement with 99DOTS; we are not able to comment on which subgroups’ outcomes benefited most from 99DOTS (or could stand to benefit more).

Other researchers have utilized qualitative and implementation science methods to identify other factors that exert a stronger influence on 99DOTS calling behaviors, including phone access (e.g., lack of phone, electricity, mobile signal, or technical literacy) as well as TB-related stigma within the household. Such factors are richly documented in a qualitative interview study of people using 99DOTS in Mumbai, Chennai, and Vellore, India [12]. Similarly, in a meta-analysis of surveyed 99DOTS deployments across Bangladesh, Tanzania, Uganda, and the Philippines, Guzman et al. find that the most common reasons for not reporting taken doses relate to battery charging, poor network connection, and forgetfulness [29]. Kiwanuka et al. report similar technical barriers to 99DOTS usage in Uganda, adding that stigma and fear of disclosure was a concern for 30% of survey respondents [30].

We also observed high variability in 99DOTS calling between sites. The scale of our study context, spanning 74 TUs, offers a new window into such site-wise variation, which has received lesser attention than person-wise variation in prior work. The variation in uptake and engagement across different areas of a single state suggests that individual providers may have chosen which and how many people to enroll in 99DOTS differently, perhaps reflecting differences in attitudes towards adopting new mHealth technologies [31]. Other location-specific factors may have also influenced usage of 99DOTS, such as physical accessibility of health facilities or cellphone coverage. Though we cannot be sure of the reasons for this site-wise variability, if this partly reflects differences in the quality of implementation of 99DOTS, there may have been diminished potential effectiveness of the technology. Additional use of implementation science or technology acceptance frameworks, such as the Consolidated Framework for Implementation Research [32] or the Unified Theory of Acceptance and Use of Technology [33], to understand drivers of local adoption of 99DOTS by healthcare providers would be valuable.

Our findings are similar to those of Cattamanchi et al., who found that 99DOTS did not improve treatment outcomes for the overall study population in Uganda. As in our study, 99DOTS’ impact may have been limited by coverage of the intervention, as only about half of people on treatment were provided 99DOTS in the intervention period. The limited reach of 99DOTS was partly driven by barriers to cellphone access among people with TB, and also by the discretion of healthcare workers in provision of the technology. As in our study, people who engaged with 99DOTS had better treatment outcomes; however, given the risk of selection bias due to non-random provision of 99DOTS, it is not possible to determine whether this effect can be attributed to 99DOTS.

Our main finding contrasts with that of Thekkur et al., who found worse TB treatment outcomes among people with HIV being treated for TB in facilities which introduced 99DOTS compared to facilities that did not pilot 99DOTS in Karnataka, India-[18]. However, the setting and implementation of 99DOTS differed greatly with the one studied here. Notably, the pilot rolled out 99DOTS simultaneously with changing the responsibility of TB care for people also living with HIV from TB program staff to ART centers under a “single window system,” plus the adoption of FDC medications. It is unclear which intervention, or possibly the complexity of rolling out several major interventions at once, may have contributed to the decrease in favorable outcomes. Subsequent research suggests that shifting TB care provision for people living with HIV from the TB program to ART centers may have increased the risk of medication non-adherence. ART centers are generally located farther away from the people they serve than the TB program’s directly observed therapy centers, such that people faced greater challenges in collecting TB medication refills on time [34].

Our analysis of the impact of 99DOTS on treatment outcomes has several limitations. First, it is possible other programmatic changes or trends could have influenced observed treatment outcomes during the study period. In particular, the Ni-kshay Poshan Yojana scheme was also introduced between our study’s pre- and post-intervention periods. While there is limited research on the impact of monetary benefits on TB treatment outcomes, we believe it is unlikely it would have worsened treatment outcomes. Second, our before and after periods were during different times of the year, and any seasonal variations (in weather, agriculture, festivals, etc.) may have affected the periods differently. That said, historical data from Himachal Pradesh suggests that treatment outcomes are comparable in each quarter of the year.^3^ Third, employing a simple pre-post design can overlook broader temporal trends in the before and after periods. While trends in treatment outcomes were not apparent in our data, there are more sophisticated techniques, including interrupted time series analysis [36], that can control for such trends. Fourth, due to partial and non-random eligibility for, and engagement with, 99DOTS, we were unable to isolate the potential effect of 99DOTS usage for people who did use 99DOTS. The recommended exclusion of certain groups from using 99DOTS, such as those with alcohol use disorder, is a confounder in the analysis, as such groups may be less likely to have favorable treatment outcomes [37, 38]. Ni-kshay administrative data does not include alcohol use disorder, as well as other characteristics (such as education and income) that could have been useful to include in the analysis. Fifth, people previously treated with TB were excluded from the analysis, in part because guidelines for treating such people were undergoing revision at the time [39]. Of TB notifications in 2017 in Himachal Pradesh, 17% were people previously treated with TB, and their rate of favorable treatment outcomes was lower compared to people treated for the first time (82% vs. 92% favorable outcomes, respectively) [21]. Excluding people previously treated with TB limits the generalizability of our results. Finally, our study design considered treatment outcomes in two buckets: favorable (cured or treatment complete) and unfavorable (died, lost to follow up, treatment regimen changed, or treatment failure). While this binarization of outcomes made it simple to state and test the key hypotheses, it may have overlooked finer-grained impacts on specific outcomes.

## Conclusions

Our study found no significant difference in the rate of favorable TB treatment outcomes before and after the state-wide rollout of 99DOTS in Himachal Pradesh. This result adds to growing evidence [19] that programs seeking to improve treatment outcomes may be wise to consider additional or alternative interventions to 99DOTS, at least as it was implemented here. Our study also contributes new insight into patterns of 99DOTS uptake, with high variability in the degree of engagement by people who used 99DOTS as well as high variability in 99DOTS usage across sites. However, our data are insufficient to understand the reasons underlying the differences in engagement, and whether steps could be taken to make 99DOTS more accessible, and more beneficial, for specific groups. In the future, it would be valuable to undertake hybrid effectiveness-implementation studies that utilize validated implementation science frameworks to understand and act on various implementation determinants. Examining factors such as cost and convenience, in addition to treatment outcomes, could also be informative for programs considering use of digital adherence technologies.

## Data Availability

The dataset has not been included as a supplement to this manuscript, because of the small possibility of compromising individual privacy. Individual researchers may be able to access the dataset, subject to the approval of the Institutional Ethics Committee at Maharishi Markandeshwar Medical College & Hospital. Inquiries can be sent via the corresponding author.

## Footnotes

1 In addition to the effective deduplication algorithms utilized by Ni-kshay, we added manual scrutiny to ensure the highest possible data quality. Of cases not already marked as previously treated, we first screened for those having (i) matching phone numbers, or (ii) fuzzy matching phone numbers and names (as per Stata’s MATCHIT module), or (iii) matching historical identifiers (from before Ni-kshay upgradation). If manual inspection of case details confirmed a duplicate or previously treated case, then we retained only one record for analysis based on a decision tree that prioritized older and more complete records.

2 Treatment regimen change can happen for multiple reasons, including: (1) lack of microbiological response at a level that does not meet definitions of treatment failure, (2) lack of clinical response motivating augmented therapy, (3) adverse reactions to medications requiring modification of the regimen, or (4) concerns for drug resistance. Given that modifications of the treatment regimen are often occurring due to clinical concerns, this is usually counted as an unfavorable outcome.

3 Data on favorable TB treatment outcomes in Himachal Pradesh is publicly available for the period from 2000 to 2008 [35]. The average treatment success is similar across quarters: 91.1% for Q1, 90.3% for Q2, 90.6% for Q3 and 91.1% for Q4. Each quarter has some data points that fall above and below the overall treatment success rate of 90.8%.
